# Epidemiology and Clinical Outcomes of Meningococcal Infections in Japan: A Nationwide Inpatient Database Study From 2010 to 2023

**DOI:** 10.2188/jea.JE20250229

**Published:** 2026-05-05

**Authors:** Satoshi Kutsuna, Hiroyuki Ohbe, Yuya Kimura, Shuhei Yokoyama, Hiroki Matsui, Kiyohide Fushimi, Hideo Yasunaga

**Affiliations:** 1 Department of Infection Control, Graduate School of Medicine Faculty of Medicine, Osaka University, Osaka, Japan; 2 Department of Emergency and Critical Care Medicine, Tohoku University Hospital, Sendai, Japan; 3 Department of Clinical Epidemiology and Health Economics, School of Public Health, The University of Tokyo, Tokyo, Japan; 4 Department of Health Services Research, Graduate School of Medicine, The University of Tokyo, Tokyo, Japan; 5 Department of General Medicine, Juntendo University Faculty of Medicine, Tokyo, Japan; 6 Department of Health Policy and Informatics, Institute of Science Tokyo Graduate School of Medicine, Tokyo, Japan

**Keywords:** meningococcal infection, epidemiology, prognosis, mortality, Japan

## Abstract

**Background:**

Meningococcal infection remains a life-threatening disease despite advances in early recognition and therapy. While the incidence of meningococcal infection is extremely low in Japan, nationwide data on its clinical outcomes have been lacking.

**Methods:**

We conducted a retrospective cohort study using the Japanese Diagnosis Procedure Combination (DPC) inpatient database from July 2010 to March 2023. Patients with a main diagnosis of meningococcal infection (International Classification of Diseases, 10^th^ Revision code A39) were included. The primary outcome was in-hospital mortality. Secondary outcomes included impaired consciousness at discharge and functional status, assessed using the Barthel Index. Multivariable logistic regression was performed to identify prognostic factors.

**Results:**

A total of 465 patients were analyzed. The median age was 55 years, and 57.0% were male. The in-hospital mortality rate was 8.2%, and 40.2% of survivors had a Barthel Index ≤90 at discharge. Early antibiotic administration did not significantly reduce mortality but was associated with improved neurological outcomes. Baseline impaired consciousness was the strongest predictor of mortality and poor functional status, while chronic comorbidities also contributed to worse outcomes. Notably, older age itself was not independently associated with mortality after adjustment for severity and comorbidities.

**Conclusion:**

Meningococcal infection outcomes in Japan are predominantly determined by the severity of illness at presentation and comorbid health conditions rather than age alone. Early antibiotic therapy improves neurological recovery but may not prevent death once critical illness is established. These findings underscore the importance of prevention through vaccination and early recognition strategies to reduce the burden of this devastating infection.

## INTRODUCTION

Meningococcal infection, caused by *Neisseria meningitidis*, is a life-threatening cause of acute bacterial meningitis and sepsis worldwide. Even with prompt antibiotic therapy, meningococcal infection carries a case-fatality rate of approximately 10–15% in developed countries.^1^^,^^2^ The global incidence of meningococcal infection is relatively low (typically <1 case per 100,000 population in high-income regions) but varies markedly by geography; historically, the highest rates occurred in sub-Saharan Africa’s “meningitis belt” during periodic epidemics.^3^^,^^4^ In Japan, meningococcal infection is extremely rare, with an annual incidence on the order of 0.01–0.04 case per 100,000 population and only a few dozens of cases reported nationwide each year.^5^^,^^6^ This low incidence may be partly explained by the lower carriage rate of *Neisseria meningitidis* observed in the Japanese population compared to Western countries, as reported in previous studies.^7^^,^^8^

Despite its infrequency, meningococcal infection remains a critical concern due to its rapid progression and severe outcomes. Known predictors of poor prognosis include advanced age and underlying comorbidities,^9^ as well as presentation with impaired consciousness or septic shock^10^^–^^12^; notably, meningococcal septic shock carries a reported fatality rate exceeding 30–50%.^13^ However, detailed epidemiologic analyses in Japan have been limited, as most studies to date rely on passive notifications or small case series.^5^^,^^6^^,^^14^

To address this knowledge gap, we conducted a nationwide study using Diagnosis Procedure Combination (DPC) inpatient database in Japan. The aim of this study was to clarify the epidemiology and clinical outcomes of meningococcal infections in Japan and to identify prognostic factors.

## METHODS

### Data source

This retrospective cohort study was conducted using the DPC database created by the DPC Study Group, a government-funded academic group responsible for creating and managing this database.^15^ This database includes data from more than 1,000 voluntarily participating acute-care hospitals, covering approximately 50% of all acute-care hospital beds across the country.^16^ It contains anonymized patient-level information including demographics, diagnoses coded according to the International Classification of Diseases, 10th Revision (ICD-10), daily procedures and medications, admission and discharge statuses, and discharge outcomes. The validity of diagnoses recorded in the DPC database has been previously evaluated, demonstrating a high specificity (93.2%) and moderate sensitivity (78.9%).^17^

### Study population

We identified hospitalized patients with meningococcal infections by selecting those with an ICD-10 code of A39 listed as the main diagnosis, admission-precipitating diagnosis, most resource-consuming diagnosis, or second most resource-consuming diagnosis between July 2010 and March 2023. We excluded patients whose diagnosis of meningococcal infection (ICD-10 code A39) was recorded as “suspected” rather than confirmed. We also excluded patients with missing outcome data. No other exclusion criteria (eg, age, treatment type, or length of stay) were applied.

### Patient characteristics

Patient characteristics included age, sex, Charlson comorbidity index, level of consciousness on admission, meningococcal disease subtype (meningitis or septicemia), interventions at admission, and early antibiotic initiation. Age was categorized into ≥65 years and <65 years. Level of consciousness was assessed using the Japan Coma Scale (JCS) at admission and was categorized into alert (JCS 0), dizziness (JCS 1–3), somnolence (JCS 10–30), and coma (JCS 100–300). ICD-10 code A39 (meningococcal infection) includes a range of clinical manifestations, such as meningococcal meningitis (A39.0) and meningococcal septicemia (A39.2–A39.4). Cases were classified as meningitis if coded as A39.0, and as bacteremia if coded as A39.2, A39.3, or A39.4. These categories were not mutually exclusive, and patients could be assigned to both groups if both codes were recorded. Other ICD-10 codes (A39.1, A39.5, A39.8, A39.9) were retained in the cohort but not categorized as either “meningitis” or “septicemia.” These codes may represent other clinical forms of meningococcal infection (eg, pneumonia, arthritis), though specific disease manifestations could not be ascertained from the administrative data. Interventions at admission included oxygen supplementation, invasive mechanical ventilation, catecholamine use, and renal replacement therapy. Antibiotic administration timing was assessed, and early antibiotic initiation was defined as administration on the day of admission. We included all eligible patients regardless of whether they received antibiotic therapy during hospitalization. Timing of antibiotic administration was assessed by hospital day, and patients with no antibiotic use were categorized separately.

### Outcomes

The primary outcome was in-hospital mortality. Secondary outcomes included decreased functional status at discharge, as indicated by a Barthel Index score of ≤90.^18^ A composite outcome, defined as in-hospital death or impaired consciousness at discharge (defined as JCS ≥1),^19^ was also evaluated.

### Statistical analysis

Descriptive statistics were used to summarize patient characteristics. Categorical variables were presented as numbers and percentages and were compared using chi-square or Fisher’s exact tests as appropriate. Continuous variables were presented as means with standard deviations (SDs) or as medians with interquartile ranges (IQRs), as appropriate, and compared using Student’s *t*-test or the Wilcoxon rank-sum test based on distribution.

Multivariable logistic regression models were used to identify independent predictors of in-hospital mortality, composite outcome, and Barthel Index ≤90. Variables entered into the models included age category (≥65 years), sex, Charlson comorbidity index, impaired consciousness at admission, meningococcal disease subtype (meningitis or septicemia), interventions at admission including oxygen supplementation, invasive mechanical ventilation, catecholamine use, renal replacement therapy, and early antibiotic initiation. Results were reported as odds ratios (ORs) with 95% confidence intervals (CIs).

All statistical analyses were performed using Stata version 17.0 (StataCorp, College Station, TX, USA). A two-sided *P*-value of <0.05 was considered statistically significant.

### Ethical considerations

This study was approved by the Institutional Review Board of the University of Tokyo (approval number, 3501-5; May 19, 2021). This study was conducted under the DPC Study Group framework using de-identified data, and the requirement for individual informed consent was waived due to the anonymous nature of the DPC data, in accordance with national ethical guidelines in Japan.

### Data sharing statement

The datasets analyzed in this study are not publicly available due to contracts with hospitals reporting data in the database.

## RESULTS

Between July 2010 and March 2023, a total of 465 patients were eligible in this study. The annual number of meningococcal infection cases remained relatively stable between 2010 and 2019, with a modest increase observed in 2015 (*n* = 60). Notably, a marked decline in case numbers was observed from 2020 onward, likely reflecting the broader epidemiological impact of the coronavirus disease 2019 pandemic. A full breakdown of annual case counts is provided in eTable 1.

The median age was 55 years (IQR, 27–73), and 57.0% were male (Table 1). The age distribution exhibited a bimodal pattern, with 13.8% of patients aged 0–9 years and 37.6% aged 65 years or older. Regarding clinical classifications, 116 patients (24.9%) were diagnosed with meningococcal meningitis (A39.0), and 273 patients (58.7%) were diagnosed with meningococcal septicemia (A39.2–A39.4). The majority of patients (75.1%) received antibiotic therapy on the day of admission. Most patients received antibiotics on the day of admission (75.1%), as summarized in Table 1. Among the 465 patients, 29 (6.2%) did not receive any antibiotics during hospitalization. These cases were retained in all analyses to avoid selection bias.

**Table 1.** Baseline characteristics and in-hospital interventions/outcomes of patients with meningococcal infection in Japan, comparing survivors vs in-hospital deaths

	Total	Survivors	In-hospital deaths	*P*-value
Patients Characteristics	*N* = 465	*N* = 427	*N* = 38	
Age, median (IQR)	55 (27–73)	53 (24–71)	72.5 (60–84)	<0.001
Age category, years, *n* (%)				
0–9	64 (13.8)	63 (14.8)	1 (2.6)	0.003
10–19	30 (6.5)	29 (6.8)	1 (2.6)	
20–29	40 (8.6)	39 (9.1)	1 (2.6)	
30–39	26 (5.6)	24 (5.6)	2 (5.3)	
40–49	47 (10.1)	47 (11.0)	0 (0.0)	
50–59	53 (11.4)	47 (11.0)	6 (15.8)	
60–69	73 (15.7)	66 (15.5)	7 (18.4)	
70–79	65 (14.0)	57 (13.3)	8 (21.1)	
80–89	62 (13.3)	52 (12.2)	10 (26.3)	
≥90	5 (1.1)	3 (0.7)	2 (5.3)	
Age >65, *n* (%)	175 (37.6)	151 (35.4)	24 (63.2)	<0.001
Male, *n* (%)	265 (57.0)	244 (57.1)	21 (55.3)	0.82
CCI, median (IQR)	0.0 (0.0–1.0)	0.0 (0.0–1.0)	1.0 (0.0–3.0)	0.002
JCS at admission, *n* (%)				
Alert (JCS 0)	293 (63.0)	283 (66.3)	10 (26.3)	<0.001
Dizziness (JCS 1–3)	98 (21.1)	80 (18.7)	18 (47.4)	
Somnolence (JCS 10–30)	39 (8.4)	33 (7.7)	6 (15.8)	
Coma (JCS 100–300)	35 (7.5)	31 (7.3)	4 (10.5)	
Meningitis, *n* (%)	116 (24.9)	112 (26.2)	4 (10.5)	0.032
Bacteremia, *n* (%)	273 (58.7)	246 (57.6)	27 (71.1)	0.11
Treatment on admission, *n* (%)				
Supplemental oxygen	82 (17.6)	65 (15.2)	17 (44.7)	<0.001
Mechanical ventilation	33 (7.1)	25 (5.9)	8 (21.1)	<0.001
Catecholamine	41 (8.8)	35 (8.2)	6 (15.8)	0.11
Renal replacement therapy	9 (1.9)	8 (1.9)	1 (2.6)	0.75
Antibiotic initiation within Day 1, *n* (%)	349 (75.1)	322 (75.4)	27 (71.1)	0.55
**Outcome**				
In-hospital death, *n* (%)	38 (8.2)	0 (0.0)	38 (100.0)	<0.001
JCS at discharge, *n* (%)				
Alert	382 (82.2)	382 (89.5)	0 (0.0)	<0.001
Dizziness	31 (6.7)	31 (7.3)	0 (0.0)	
Somnolence	7 (1.5)	7 (1.6)	0 (0.0)	
Coma	7 (1.5)	7 (1.6)	0 (0.0)	
In-hospital death	38 (8.2)	0 (0.0)	38 (100.0)	
Composite Outcome, *n* (%)	83 (17.8)	45 (10.5)	38 (100.0)	<0.001
Functional Status at discharge (Barthel Index <90), *n* (%)	158 (40.2)	120 (33.8)	38 (100.0)	<0.001
Total hospitalization cost, thousand Japanese yen, median (IQR)	683 (351–1,531)	650 (349–1,442)	1,285 (410–2,558)	0.037
Length of hospital stay, days, median (IQR)	16 (8–33)	15 (8–33)	19 (5–46)	0.88
ICU/HCU admission, *n* (%)	113 (24.3)	96 (22.5)	17 (44.7)	0.002
Treatment during hospitalization, *n* (%)				
Supplemental oxygen	156 (33.5)	132 (30.9)	24 (63.2)	<0.001
Mechanical ventilation	55 (11.8)	41 (9.6)	14 (36.8)	<0.001
Catecholamine	71 (15.3)	51 (11.9)	20 (52.6)	<0.001
Renal replacement therapy	31 (6.7)	22 (5.2)	9 (23.7)	<0.001
Blood transfusion	62 (13.3)	48 (11.2)	14 (36.8)	<0.001
Amputation, *n* (%)	2 (0.4)	2 (0.5)	0 (0.0)	0.67

CCI, Charlson Comorbidity Index; HCU, high care unit; ICU, intensive care unit; IQR, interquartile range; JCS, Japan Coma Scale; SD, standard deviation.

In-hospital deaths were defined as patients who died during the index hospitalization.

In-hospital mortality was 8.2% (*n* = 38). The composite outcome of in-hospital death or neurological impairment at discharge, defined as a JCS score of ≥1, occurred in 17.8% of patients. Among survivors, 40.2% had a Barthel Index ≤90.

Multivariable logistic regression analysis identified several independent predictors of in-hospital mortality (Table 2). Higher Charlson comorbidity index was significantly associated with higher in-hospital mortality (OR 1.58; 95% CI, 1.14–2.17; *P* = 0.005). Impaired consciousness on admission, particularly JCS 1–3; dizziness (OR 4.75; 95% CI, 1.90–11.84) and JCS 10–30; somnolence (OR 4.93; 95% CI, 1.44–16.89), was also associated with higher in-hospital mortality. Oxygen supplementation on the day of admission was another significant predictor (OR 5.23; 95% CI, 2.17–12.63), and the need for invasive mechanical ventilation on the day of admission was associated with markedly increased odds of death (OR 22.75; 95% CI, 5.07–102.12). In contrast, the use of catecholamines, renal replacement therapy, and early antibiotic initiation were not significantly associated with in-hospital mortality.

**Table 2.** Multivariable logistic regression analysis of factors associated with outcomes of meningococcal infection

Predictor	In-hospital mortality OR (95% CI)	Composite outcome OR (95% CI)	Poor functional status OR (95% CI)
Age ≥65 years	1.72 (0.74–4.00)	3.44 (1.84–6.45)	3.79 (2.28–6.29)
Male sex	0.74 (0.34–10.64)	1.21 (0.67–2.19)	0.76 (0.47–1.24)
Charlson Comorbidity Index	1.58 (1.14–2.17)	1.15 (0.89–1.47)	1.10 (0.89–1.37)
JCS 1–3 (dizziness)	4.75 (1.90–11.84)	7.51 (3.74–15.09)	2.44 (1.33–4.46)
JCS 10–30 (somnolence)	4.93 (1.44–16.89)	10.83 (4.36–26.89)	7.53 (3.10–18.69)
JCS 100–300 (coma)	0.87 (0.21–3.60)	7.11 (2.71–18.65)	2.41 (0.98–5.91)
Meningococcal meningitis	0.38 (0.10–1.49)	0.42 (0.17–1.01)	0.29 (0.13–0.64)
Meningococcal septicemia	1.59 (0.60–4.21)	0.73 (0.36–1.49)	0.73 (0.38–1.39)
Oxygen therapy on admission	5.24 (2.17–12.63)	2.27 (1.18–4.39)	1.78 (0.97–3.28)
Endotracheal intubation on admission	22.75 (5.07–102.12)	6.54 (2.00–21.46)	5.57 (1.91–16.19)
Catecholamine use on admission	0.53 (0.13–2.23)	0.74 (0.24–2.30)	0.81 (0.33–2.02)
RRT on admission	0.81 (0.07–8.93)	0.47 (0.07–3.32)	6.72 (0.71–63.96)
Antibiotic initiation within Day 1	0.60 (0.25–1.44)	0.51 (0.26–0.98)	0.78 (0.45–1.36)

CI, confidence interval; JCS, Japan Coma Scale; OR, odds ratio; RRT, renal replacement therapy.

Shown are adjusted ORs with 95% CIs for three outcomes: in-hospital mortality, composite outcome (in-hospital death or neurologic impairment at discharge), and poor functional status (Barthel Index ≤90 at discharge).

Analysis of the composite outcome of in-hospital death or neurological impairment at discharge revealed that older age (≥65 years) (OR 3.44; 95% CI, 1.84–6.45), impaired consciousness at admission (dizziness: OR 7.51; 95% CI, 3.74–15.09; somnolence: OR 10.83; 95% CI, 4.36–26.89), oxygen supplementation (OR 2.27; 95% CI, 1.18–4.39), and invasive mechanical ventilation (OR 6.54; 95% CI, 1.99–21.46) were independently associated with worse outcomes. Early initiation of antibiotics within day 1 was associated with a lower risk of the composite outcome (OR 0.51; 95% CI, 0.26–0.98).

Analysis of the Barthel Index of ≤90 at discharge demonstrated that older age (≥65 years) (OR 3.79; 95% CI, 2.28–6.29), impaired consciousness on admission (dizziness: OR 2.44; 95% CI, 1.33–4.46; somnolence: OR 7.53; 95% CI, 3.03–18.69), and endotracheal intubation (OR 5.57; 95% CI, 1.91–16.19) were significant predictors of poor functional outcomes. The presence of meningococcal meningitis (A39.0) was inversely associated with a Barthel Index ≤90 (OR 0.29; 95% CI, 0.13–0.64).

## DISCUSSION

This study clarified the national epidemiology and clinical outcomes of meningococcal infections in Japan and identified prognostic factors, highlighting that disease severity and comorbidities were the primary determinants of in-hospital mortality, while early antibiotic therapy was associated with improved neurological recovery without significantly lower mortality.

The 8.2% in-hospital mortality observed in our nationwide DPC-based study is notably lower than the case-fatality rate reported by the National Institute of Infectious Diseases for invasive meningococcal disease in Japan (∼12%).^5^ This can be because the National Institute of Infectious Diseases data include only culture-confirmed invasive meningococcal disease (eg, isolation from blood or cerebrospinal fluid), whereas our analysis encompassed a broader spectrum of meningococcal infections (including non-invasive presentations, such as meningococcal pneumonia). This broader inclusion may capture milder cases that would be underreported in the surveillance system, thereby increasing the denominator and yielding a lower overall mortality. Indeed, under-ascertainment of patients with less severe invasive meningococcal disease in passive surveillance has been noted as a concern in settings with traditionally low incidence.^20^ Differences in case mix and hospital setting could also contribute. That is, our dataset of hospitalized patients may include prompt-treated cases and those with non-invasive infection, whereas the National Institute of Infectious Diseases surveillance inherently focuses on the most severe, lab-confirmed episodes of invasive meningococcal disease. Notably, the mortality rate and prognostic factors identified in our study are in line with reports from other regions. In Western countries, case-fatality of meningococcal infection generally ranges around 5–15% even with modern care,^21^ and risk of death is known to rise significantly with advanced age, comorbid conditions, and indicators of severe disease at presentation. For example, a meta-analysis found that invasive meningococcal disease mortality risk was higher in adults than in young children and was higher in infections caused by serogroup C relative to serogroup B.^22^ In the present study, impaired consciousness was associated with worse outcome. This finding is consistent with that in a previous study that showed fulminant meningococcemia or meningitis with shock markedly elevated mortality risk.^23^ Meanwhile, published data from Asia similarly showed substantial meningococcal infection fatality and analogous risk factors. That is, in China and Korea, reported case-fatality rates ranged from approximately 5% to 12% in recent years, and higher mortality was associated with patient age, serogroup, and disease severity.^20^^,^^23^ Overall, the present study showed the mortality rate and its key determinants (older age, underlying illness, and impaired consciousness) in Japan were broadly consistent with those in other countries, although Japan has unique epidemiology of meningococcal infection, characterized by an older patient age distribution and a predominance of serogroup Y cases.^5^

This study found that in-hospital mortality from meningococcal infection was primarily driven by baseline illness severity and patient comorbidities, while early antibiotic administration was associated with better neurological outcomes but was not significantly associated with lower mortality. In acute bacterial meningitis, each hour of treatment delay markedly increases the risk of death and of long-term sequelae,^24^^,^^25^ whereas prompt antibiotic therapy improves outcomes.^26^ Consistent with these patterns, our results showed that baseline severity of illness was a key determinant of outcome. Although previous studies have shown that even short delays in antibiotic therapy can significantly increase mortality in patients with bacterial meningitis, our analysis did not find a significant association between early antibiotic administration and in-hospital death. This discrepancy may reflect several factors, including the limited temporal resolution of the DPC database (which records antibiotic initiation only by calendar day), the potential for irreversible disease progression before admission, and the heterogeneity of clinical syndromes (eg, meningitis, septicemia, pneumonia). Thus, considerable heterogeneity in actual time to therapy likely existed within the same calendar day. Furthermore, the strongest predictor of mortality in our study was baseline neurological status, suggesting that once fulminant disease has developed, the therapeutic window may already have passed. These findings emphasize that early recognition and intervention before progression to severe illness may be more impactful than antibiotic timing alone, particularly in settings where precise onset-to-treatment intervals are not captured. That is, impaired consciousness on admission was the strongest predictor of mortality and poor recovery. This aligns with previous reports that showed neurological status at presentation was a critical prognostic factor in both pediatric and adult meningitis.^12^^,^^27^ Chronic comorbid conditions were also associated with worse outcomes, likely due to reduced physiological reserve and possible delays in diagnosis; such conditions predispose individuals to invasive meningococcal disease.^28^

Our analysis did not find advanced age to be an independent risk factor for mortality after adjusting for illness severity and comorbidities, unlike some prior studies. Typically, older adults have substantially higher case-fatality rates—nearly double those of younger patients—in invasive meningococcal disease.^21^ In our cohort, however, once we accounted for severity and comorbidities, age conferred no additional risk. This discrepancy may indicate that functional status at presentation is more important than chronological age for prognosis.^22^ It is also possible that our sample included relatively few elderly patients or that uniformly aggressive care across ages in Japan mitigated age-related differences. This finding should be interpreted with caution, as limited power or residual confounding might obscure an age effect.

Once fulminant meningococcal disease is established, its course is difficult to alter, underscoring the importance of prevention and early intervention. Meningococcal vaccination has significantly reduced disease incidence and mortality in high-income countries.^29^ In Japan, where cases are extremely rare, targeted immunization of high-risk groups (eg, individuals with asplenia or complement deficiencies) could further reduce the incidence of disease.^28^ Additionally, improving public and provider awareness of meningococcal symptoms is crucial to facilitate prompt recognition and treatment; increased awareness, alongside vaccination, has been linked to better outcomes.

This study has several limitations. First, case identification relied on administrative coding using ICD-10 code A39, introducing the possibility of misclassification. Some patients may have been incorrectly classified as meningococcal infection, while true cases may have been missed if not coded properly. Although previous studies have validated the overall reliability of the DPC database,^17^ administrative codes are inherently less accurate than microbiological confirmation and may introduce bias in epidemiologic estimates. In addition, a subset of patients classified under codes such as A39.8 or A39.9 may have had milder or atypical forms of meningococcal infection (eg, pneumonia without bacteremia), which could influence outcome distribution but could not be reliably distinguished in the administrative dataset. Second, the DPC database lacked detailed clinical and microbiological information, such as cerebrospinal fluid analysis results, pathogen serogroup identification, antimicrobial susceptibility test results, or the exact timing of antibiotic administration relative to symptom onset. The inability to assess bacterial resistance profiles limited our understanding of treatment adequacy and potential impacts of resistant strains on outcomes. Moreover, the absence of granular clinical data restricted our ability to fully adjust for disease severity or to perform subtype-specific prognostic analyses. Third, unmeasured variables, such as pre-hospital delays, variations in clinical management, or differences in host immune response, may have influenced prognostic factor analyses. Finally, we did not adjust for corticosteroid use in our analysis. Unlike in pneumococcal meningitis, the benefit of adjunctive corticosteroid therapy in meningococcal meningitis has not been well established, and it is not routinely recommended. Furthermore, our cohort included patients with a wide range of clinical presentations—such as meningitis, septicemia, and pneumonia—caused by *Neisseria meningitidis*. Therefore, the indication and timing of corticosteroid administration would have been highly variable and non-standardized across patients. Although some patients may have received corticosteroids, we were unable to account for their potential effects on outcomes. Additionally, certain clinical variables used in this study warrant further discussion. Functional outcomes, such as the Barthel Index and JCS, were assessed at discharge but may be influenced by evaluator subjectivity or institutional protocols. Similarly, medical interventions, such as oxygen therapy or mechanical ventilation, were derived from administrative billing data and may not fully reflect the clinical indications, timing, or severity. These limitations are inherent to the use of administrative databases and may have introduced residual confounding in the outcome analyses.

In conclusion, this nationwide study clarified the epidemiology and clinical outcomes of meningococcal infections in Japan and identified key prognostic factors. Disease severity at presentation and comorbidities were the strongest determinants of in-hospital mortality, while early antibiotic administration was associated with better neurological outcomes. These findings underscore the importance of early recognition, aggressive supportive care, and preventive strategies such as targeted vaccination to reduce the burden of this rare but severe disease.

## SUPPLEMENTARY MATERIAL

eTable 1. Annual cases of meningococcal infection in this study
