# Supplementary material for: Epidemiology and Clinical Outcomes of Meningococcal Infections in Japan: A Nationwide Inpatient Database Study From 2010 to 2023
Source: J Epidemiol. 2026 May 5;36(5):162–7. doi: 10.2188/jea.JE20250229 (PMC13085648; doi:10.2188/jea.JE20250229)
Supplement: Supplementary file 1 [file je-36-162-s001.pdf]

eTable 1. Annual cases of meningococcal infection in this study

Fiscal Year	Number of Cases
2010	37
2011	52
2012	41
2013	33
2014	37
2015	60
2016	34
2017	40
2018	46
2019	36
2020	14
2021	19
2022	16
